# Miscellaneous Notes

**Published:** 1872-08

**Authors:** 


On the Diagnosis of Typhoid Fever in its Early Stages.—The great importance of the following remarks, taken from a clinical lecture on this subject by Dr. P. W. Latham, Cambridge, England (*London Lancet*, June, 1872), although overlooked at the proper time, causes us to republish them, and to recommend them to the serious consideration of our readers:

"From the information the thermometer gives me, I can fully indorse the following statement: 'The physician who judges of fever cases without taking note of the temperature, is like a blind man trying to find his way. With much practice and great intelligence, the blind man may succeed; but he will more frequently fail, and always do, only with great difficulty and unsatisfactorily, what to him who sees, requires no effort.'

"Let me show you how far this is true. During the first four or five days, the general symptoms which may then, as I have told you, accompany the disease—viz.. the rigor, the languor and feebleness, headache, epistaxis, giddiness, pain in the back and aching of the limbs, the appearance of the tongue, the state of the bowels, the condition of the urine, etc.—may not be very distinct, or any of these morbid symptoms may be entirely absent. In a considerable number of cases, in fact, it would be impossible for you to say, without using the thermometer, whether the patient were suffering from typhoid fever or not. But the thermometric course of the disease at this time, unless it supervenes on some other malady, is very regular; and by taking the temperature at eight A.M. and six P.M. for three days, the presence of typhoid fever may be decided. On the other hand, one single observation may, with very great probability, negative the existence of the disease.

"The following is the formula (from Wunderlich) of this initial stage:

| | Morning. | | Evening. | |
|---|---|---|---|---|
| First day | 98.6° | Fahrenheit. | 100.4° | Fahrenheit. |
| Second day | 99.4° | " | 101.4° | " |
| Third day | 100.4° | " | 102.6° | " |
| Fourth day | 101.6° | " | 104° | " |

"If, then, a person, previously quite well, feels uneasy, perhaps has a rigor, and in the evening we find his temperature about 100.4° or 101°F., falling the next morning about a degree, rising again in the evening, and approximately following the above course, the disease may be diagnosed with tolerable certainty.

On the other hand, the disease is not typhoid fever if (1) on the second, third, or fourth evening, the temperature approximates even to the normal (98.6°F.); (2) if during the first two days, the temperature rises to 104°F.; (3) if between the fourth and sixth days, the evening temperature of a person under middle age does not reach 103°; (4) if the temperature on two of the first three evenings is the same; or (5) if it is the same on the second and third mornings. From the fourth to the tenth day, the evening temperatures are tolerably uniform, the highest being most generally on the evenings of the fourth, fifth, or sixth days, and reaching from 104° to 105.5°F., or even higher. The morning temperatures are from 1° to 2.6°F. lower than the evening ones; on the fifth, sixth, and seventh days, the variations between the morning and evening temperatures being less than take place from the sixth or seventh to the ninth or tenth days. During this period (from the fourth to the tenth or twelfth day), if the general symptoms are obscure, an absolute diagnosis may not be readily made, and the disease may be confounded with several others, unless thermometric observations extend over several days. Here, again, the thermometer gives us then very definite information. Wunderlich tells us—'That if in a youth or individual of middle age, who was previously well, an illness has continued from five to ten days, and we find evening temperatures of 103.4° to 105°F., or a little lower, alternating with morning temperatures of 1.4° to 2.6°F. lower, and without any other disorder showing itself to explain the elevation, and without the patient previously being subject to gross neglect, then we are justified in positively diagnosing the disease as typhoid fever.' There is at the present time in the hospital a girl in whose case the application of this law materially helped us to arrive at a correct diagnasis; from the ninth to the twenty-first days of the disease, as you see from the chart, her evening temperatures mostly reached 103° to 103.8°F., with some morning temperatures 1° and some 2°

lower. This, excepting small successive crops of rose spots, was the only distinct evidence of the disease; the other symptoms—the condition of the tongue, the character of the evacuations, etc.—not being at all characteristic. The normal temperature in this case was not reached until the thirty-fifth day."

On the Use of Pepsine Wine in the Artificial Feeding of Infants.—Dr. W. Jackson Cummins (*Dublin Journal of Medical Science*, February) made an interesting communication on this subject to the Cork Pathological and Medico-Chirurgical Society. "The value of pepsine," he remarked, "in those forms of dyspepsia attended by a deficient secretion of gastric juice, is so well known and generally understood, that it is unnecessary for me to trespass on the time of the Society by more than an allusion to them. In the diseases of children, however, and especially as a substitute for a wet-nurse, when a mother is unable or unwilling to suckle her own child, the benefit of this valuable aid to digestion is not, I believe, as generally known, although allusions to it are to be found in medical essays. * * * *

"There is nothing, of course, like a good breast of milk for an infant, if it can be had; and in the 'good old times,' when the peasantry and small farmers lived on potatoes and milk, without stimulating their nerves with strong tea, nor their brains with penny-a-liner's novels, there was an ample field for the selection of a foster parent; but now, even when the *rara avis*, a good nurse, is procured, she is so independent, and knows her power so well, that any caprice must be humored, and she is always ready to throw up her situation or neglect her charge. A wet-nurse is, then, an admitted torment, and a balance struck between its advantage and disadvantage is generally against the former.

"Artificial feeding by bottle is a great improvement upon the old system of spoon-feeding, as the act of suckling stimulates the salivary glands and insures due insalivation, which is an important part of infantile digestion. With such an aid, the stomach of most *human* infants is vigorous enough to fall into the way of digesting *cow's* milk, properly diluted, and mixed with sugar and cream to assimilate the proportion of its constituents to human milk; but besides the relative excess of casein and albumen contained in cow's milk when compared with human, the coagulum of the latter is 'soft, flocculent, and not so thoroughly separated from the other elements of the fluid as the firm, hard curd of cow's milk is from the whey in which it floats.'—*West.* And when we reflect that the digestive organs of the *human* infant are found to digest human milk, and the force of its gastric juice proportioned to the solution of its soft, flocculent coagulum, we can understand why the solvent power of its gastric juice is sometimes unequal to redigesting the firm curd of cow's milk. When such is the case, acetous fermentation is quickly set up, offensive gases distend the stomach and taint the breath, vomiting and diarrhœa set in, and in process of time the little patient sinks into a miserable state of marasmus, and dies.

"The remedy for this state of things is simple; for although we can not change the elementary composition of the milk we have to use, we can introduce into the infant's stomach a digestive power proportioned to the food it has to use—the organic principle of digestion taken from the stomach of the calf.

"It is now many years since I first applied this simple theory to practice in the case of one of my own children, who, when about three or four months old, was reduced to a condition of marasmus by vomiting and diarrhœa, due to imperfect digestion of cow's milk. I ordered him fifteen or twenty drops of pepsine wine, to be given immediately before or after each meal. Soon after commencing it he began to improve, and by degrees, all bad symptoms vanished, and nutrition was quite restored. The pepsine was continued until he was nearly two years old, and he throve at least as well as if he had been wet-nursed; other treatment, of course, preceded and accompanied the use of pepsine, but it was not until the latter was commenced that improvement took place.

"Shortly after, a child born in England, and bottle-fed, was brought over to this country when about six months old; he also was suffering from infantile dyspepsia, and was pining away in a listless, apathetic state, quite indifferent to surrounding objects, and appearing as if he would lapse into idiocy from from mal-nutrition of the nervous centers. I immediately ordered him pepsine wine, which produced such beneficial effects that, after it had been continued about twelve months, he had become a bright, intelligent, well-nourished child.

"Since then, I have never recommended a wet-nurse, and have used pepsine wine largely in dispensary, hospital and private practice, and have seen many apparently hopeless cases recover under its use."—*Half-Yearly Abstract.*

Treatment of Small-Pox by Vaccination and Injections of Lymph.—Dr. Turley, of Edinburgh (*London Lancet,* August, 1872), has, by a large experience in the treatment or cow-pox, arrived at the conclusion that vaccination is not only of prophylactic but curative value in the treatment of this disease. While he finds the usual method of vaccination sufficient in children to produce this latter effect, it does not answer the purpose in grown persons, and he relies on subcutaneous injections of lymph. These injections produce an abortive effect on the eruptions, and this effect is so much more certain, the earlier in the disease the remedy is used. The vesicles do not attain their full size—do not become pustular, and dessicate earlier.

Preparation of Kumyss from Condensed Milk.—The very favorable results which Russian physicians have obtained by the use of the genuine kumys, in the steppes of that country, as a dietetic in the treatment of pulmonary consumption and chronic derangements of nutrition, have caused an imitation of this substance in Germany and Switzerland, by the use of which the same favorable results have been obtained. Dr. Carl Schwalbe, at Zürich, furnishes the following very simple method of preparing an article of kumyss, which fulfills all the reasonable demands to be made on a dietetic remedy: 100 centigrammes of condensed milk are dissolved in a small quantity tity of cold water; 1.0 gramme of lactic acid which has been previously dissolved in 0.5 gramme of citric acid, and 15.0 gramme of good rum, are added, and the whole is diluted with enough water to make in all 1,000 to 1,500 centigrammes of common fluid. This mixture is put in a Liebig's bottle, and impregnated with carbonic acid gas. This is kept in a warm room; and if, on testing it, extensive development of foam and fine granular coagulation have commenced, it is ready for use. It remains fit for use about one week. If it is used permanently, it is, of course, necessary to have two bottles. The advantages of this method of using kumyss are the following:

1. The relative quantities of the different component parts can be changed to suit the condition of each peculiar case; and also the peculiar taste of each individual can be satisfied—a matter of some importance in a long-continued course of treatment. Thus the rum can be substituted by other liquors—arrack, cognac, etc.; and instead of citric acid, small quantities of muriatic or phosphoric acid, etc., can be used.

2. The casein of this kumyss is divided unusually fine (while that of the genuine kumyss of Davose remains in more or less large lumps), and is, therefore, very easily digested.

3. It can be easily prepared everywhere, even on land and sea-journeys.

4. Its taste is much more agreeable than that of the original kumyss, and the price much lower.—*Berliner Klinische Wochenschrift*, 25, 1872.

Ammonia Hypodermically in Asphyxia.—The value of the injection of ammonia, as recommended by Prof. Halford, in case of snake-bite and suspended animation, has been again demonstrated. A lady in Melbourne recently swallowed by accident an ounce of Brown's chlorodyne, which is a mixture of chloroform, morphia, and prussic acid. When seen by her medical attendant, she was, as he imagined, on the point of death—cold, insensible to everything, and giving only occasional gasps as signs of breathing. Recollecting a former case, in which a young man who had taken chloroform was revived after death had apparently occurred, the doctor mixed half a drachm of the liq. ammon. fort. with one and a half of water, and within the space of one minute injected the whole into a vein of the arm. In a few minutes the pulse returned, the breathing became natural, and by twenty minutes the whole body had regained its natural warmth; but perfect consciousness did not return for some hours afterward. The patient made a rapid recovery. Two further instances have also been reported, in which the timely use of the injection saved the victims of snake-bite from the death which threatened them.—*Melbourne Argus, from the Medical Cosmos.*

Substitute for the Ophthalmoscopic Mirror.—The ophthalmoscope having come into general use in medicine, it may save considerable embarrassment in choosing from among the vast variety of instruments offered, to point out that we all possess one already in our watch-pockets—viz., a watch-glass. Any transparent reflecting body is an ophthalmoscope, and, with the ordinary lamp, quite enough light is returned from the concavity of a watch-glass to display all the details of the fundus of the eye. It is used in the same manner as the mirror, but possesses this advantage, that (in viewing the inverted image) it may be held at any convenient distance from the obesrver's eye, so long as he looks through it. Therefore, if more light be desired, the watch-glass may be approximated to the patient's eye till all its reflection is concentrated on his pupil. Atropine is not required except in special cases, as there is no glare to provoke contraction of the pupil. Any watch-glass will do provided the glass be (as it is in almost all of them) equally thick throughout, so as not to distort the image seen through it. Improved forms will, of course, at once occur to the reader—*e. g.*, with a central convexity to enlarge the image or concavity to correct myopia; but to discuss these would be foreign to my purpose, which is merely to point out the use of what we have already got—the employment of which, I may add, was suggested to me by the reports of some of Mr. Carter's admirable experiments.—*Dr. J. S. Torrop, in London Lancet.*

Use of Nux Vomica in Certain Neuroses of Organic Life.—M. Brugnoli has employed nux vomica successfully in the nervous movements of pregnancy, gastralgia, dyspepsia, hypochondriasis, nervous palpitations of the heart, nervous and periodic cough, asthma, and finally, in albuminuria. This remedy acts either on the pneumogastric, or on the great sympathetic, or on the spinal cord. He records a case of a lady affected with severe cough recurring every evening and lasting throughout the night, who was cured in two days by the use of nux vomica. Another patient was affected every evening with violent cough, accompanied by catarrhal expectoration, and was also cured in two days by the use of the alcoholic extract of nux vomica mixed with the extract of gentian. Cough may always be allayed by this means, whether it be caused by bronchitis, by pneumonia, by pulmonary phthisis, or by emphysema. It proves a useful remedy also in cases of cardiac pulsations, and in irregular or too frequent action of the heart. In albuminuria, M. Brugnoli thinks the administration of nux vomica has retarded its progress to some extent, especially in cases of scarlatinal albuminuria.—*The Practitioner, from Braithwaite's Retrospect.*

On the Exhibition of Calomel.—The changes which preparations containing calomel undergo by being kept are discussed by Vulpius in an article we translate from the *Allgemeine Medicinische Centralzeitung*, March, 1872:

In mixtures of calomel with white sugar, magnesia, or sugar of milk, no signs of corrosive sublimate can be found even after three months. Traces of sublimate do appear in a mixture of calomel, bicarbonate of soda, and sugar of milk, kept an equal time. Decided quantities were present in a mixture of calomel, bicarbonate of soda, and cane sugar, preserved the same period and exposed to moisture. The last-mentioned is the only preparation which he decides should not be kept on hand for pharmaceutical purposes, but should be freshly-mixed when wanted. The occasional change of small portions of calomel into corrosive sublimate in the stomach or bowels he does not consider of any serious moment, and calls attention to the opinion lately expressed by Dr. Zeroni, of Manheim, who attributes the cure of a case of cerebro-spinal meningitis by large doses of calomel to this very change, in some degree.—*Half-Yearly Compendium.*

